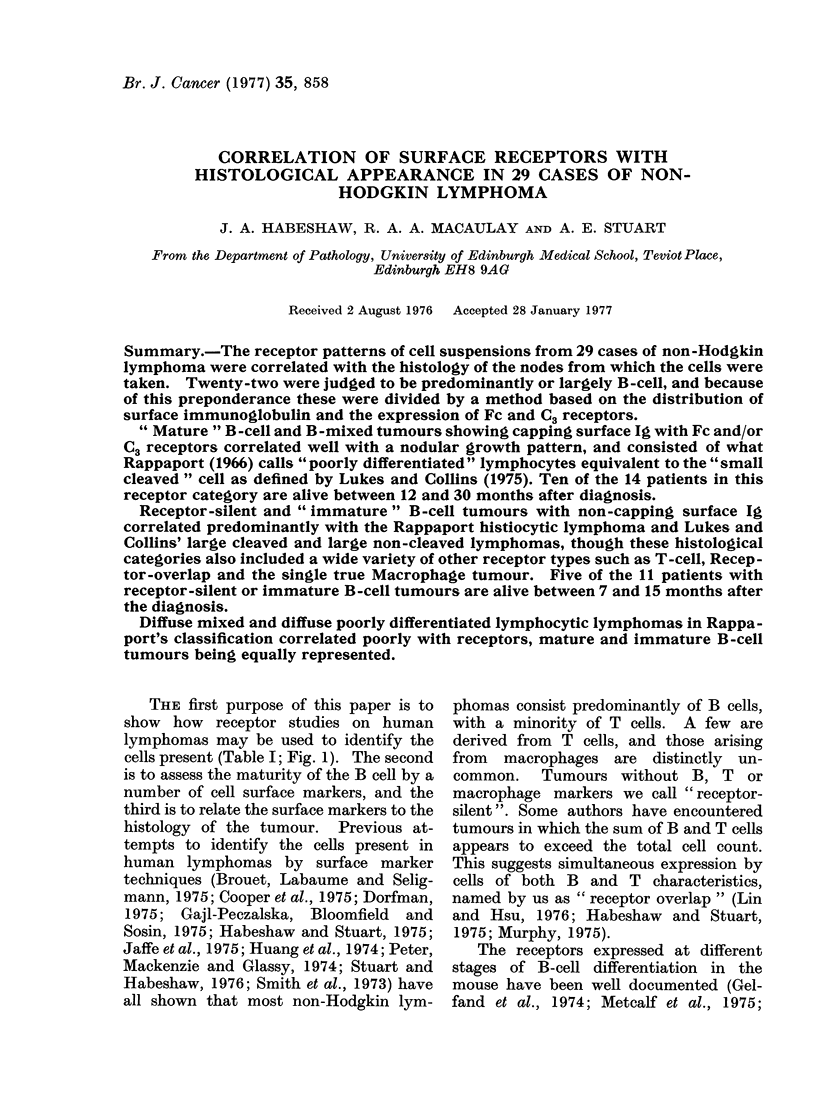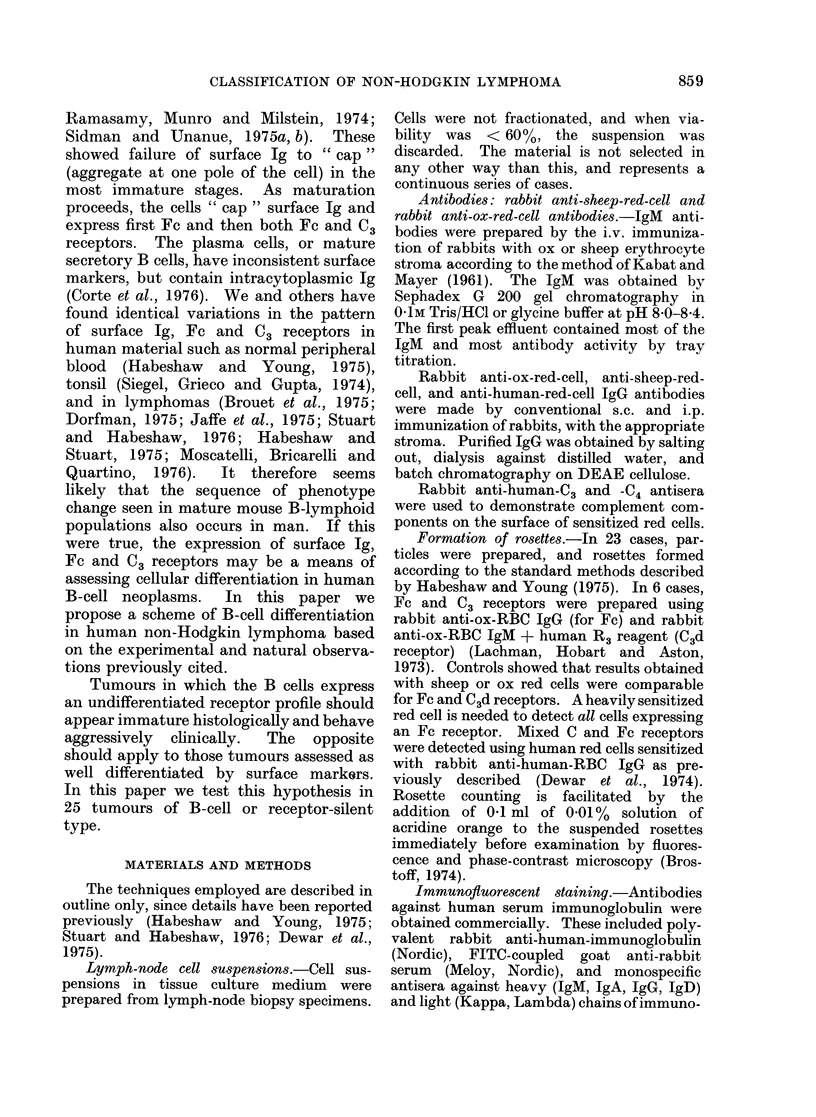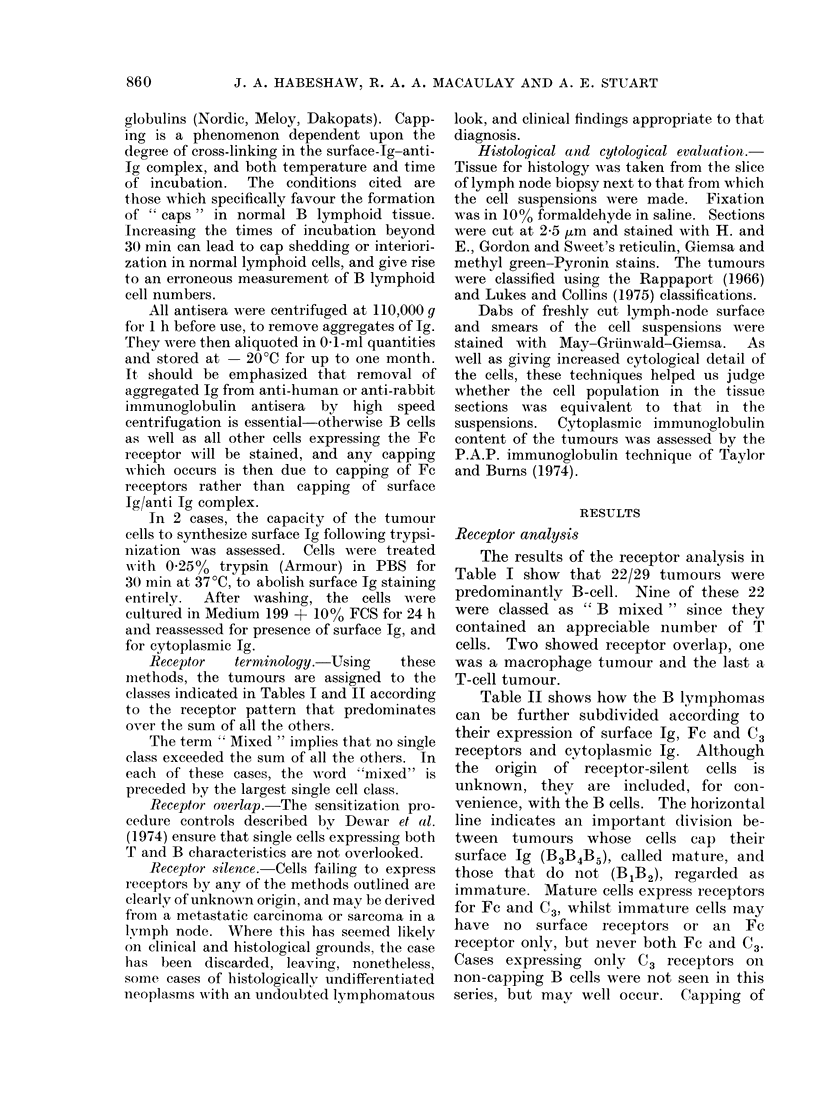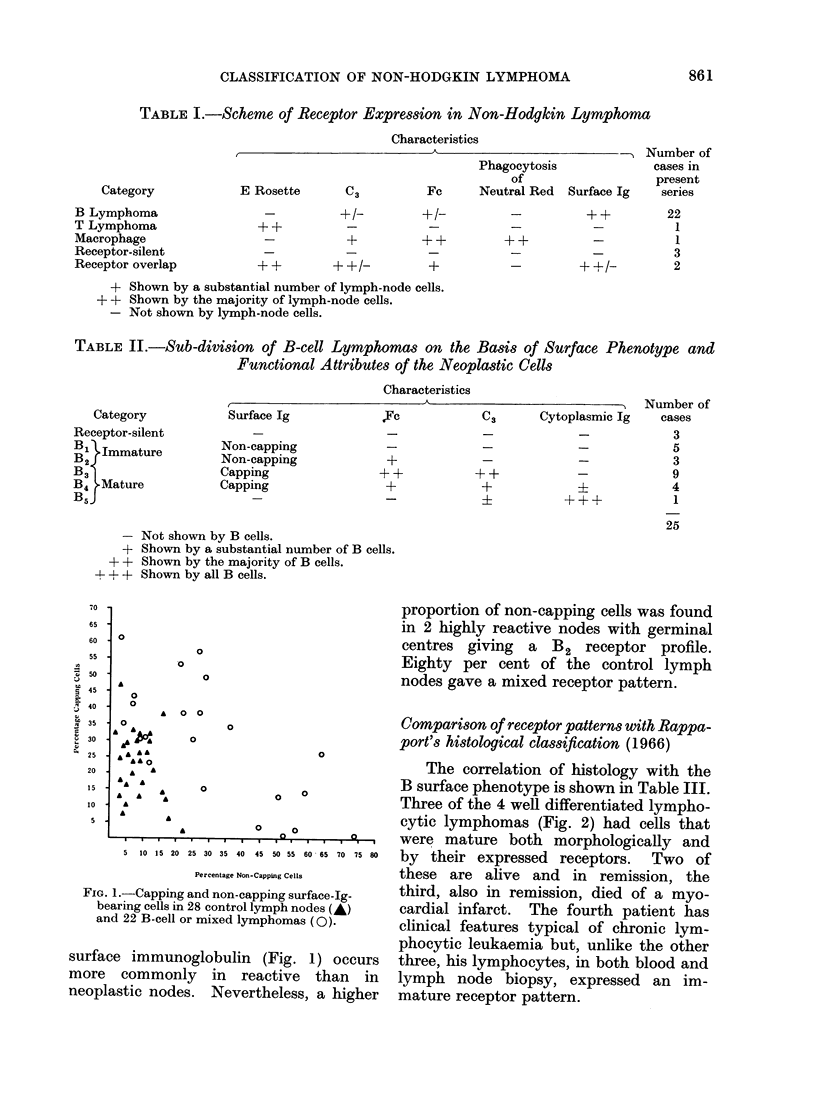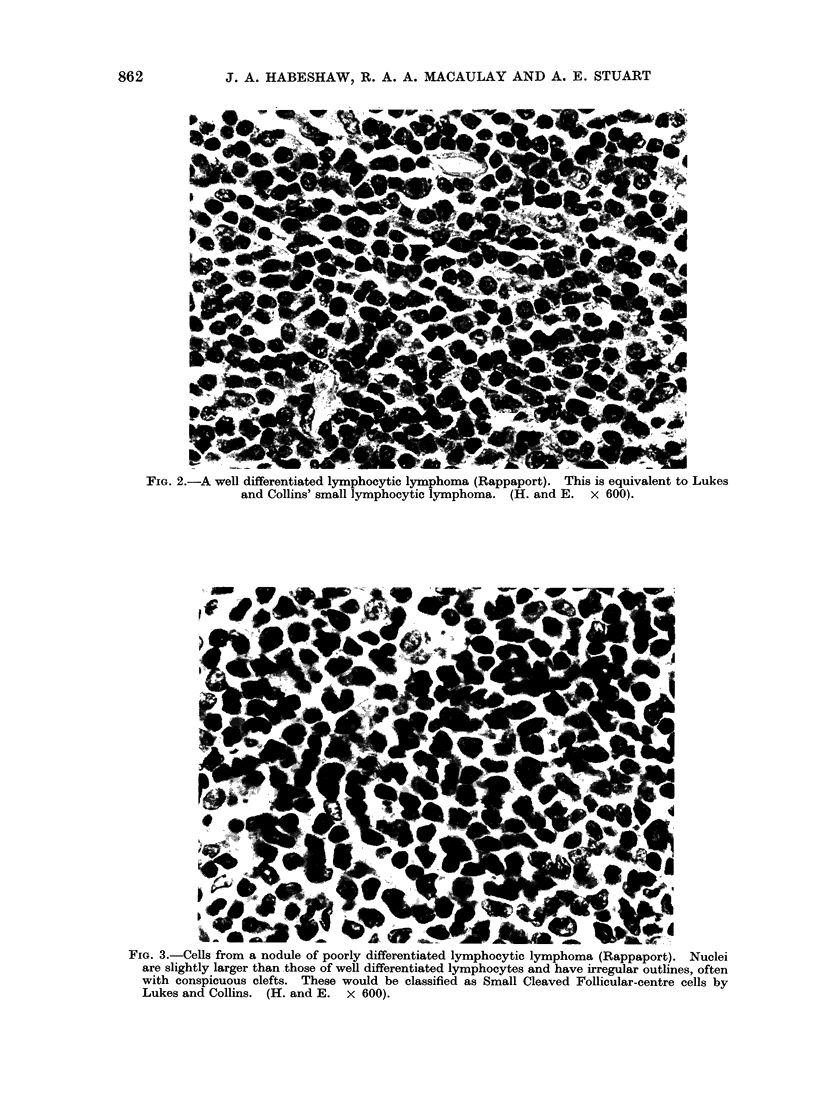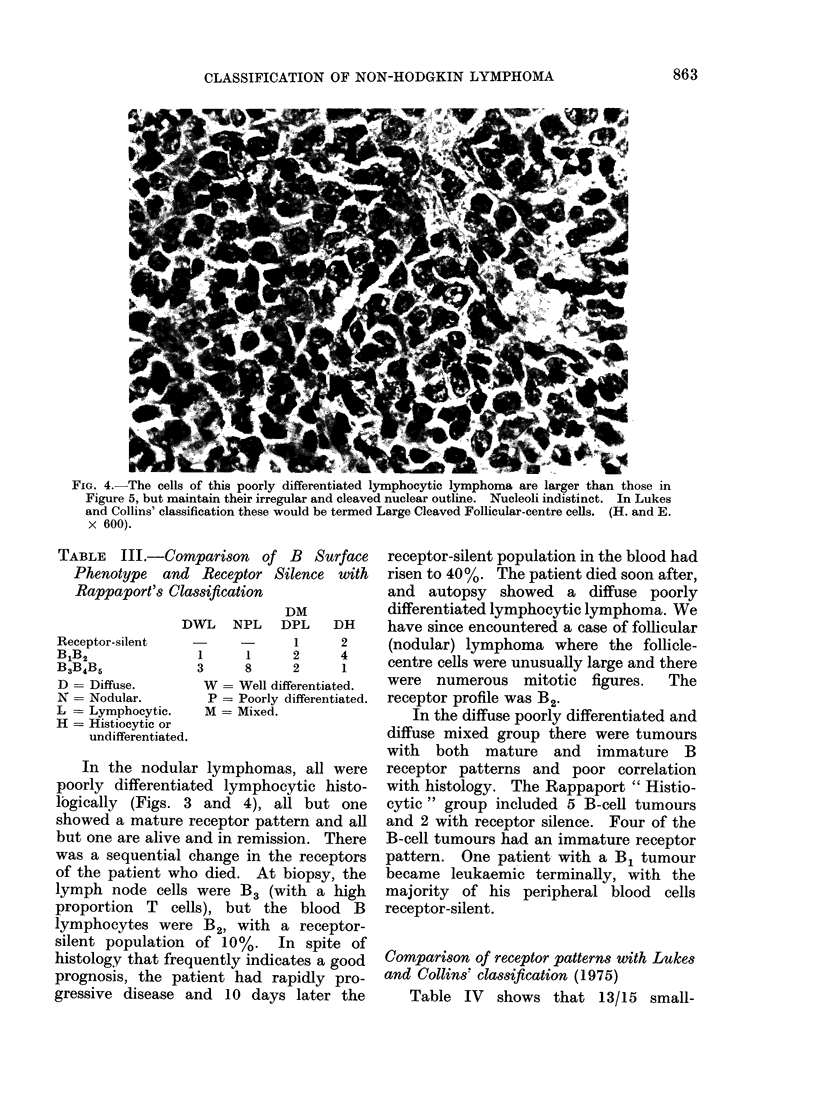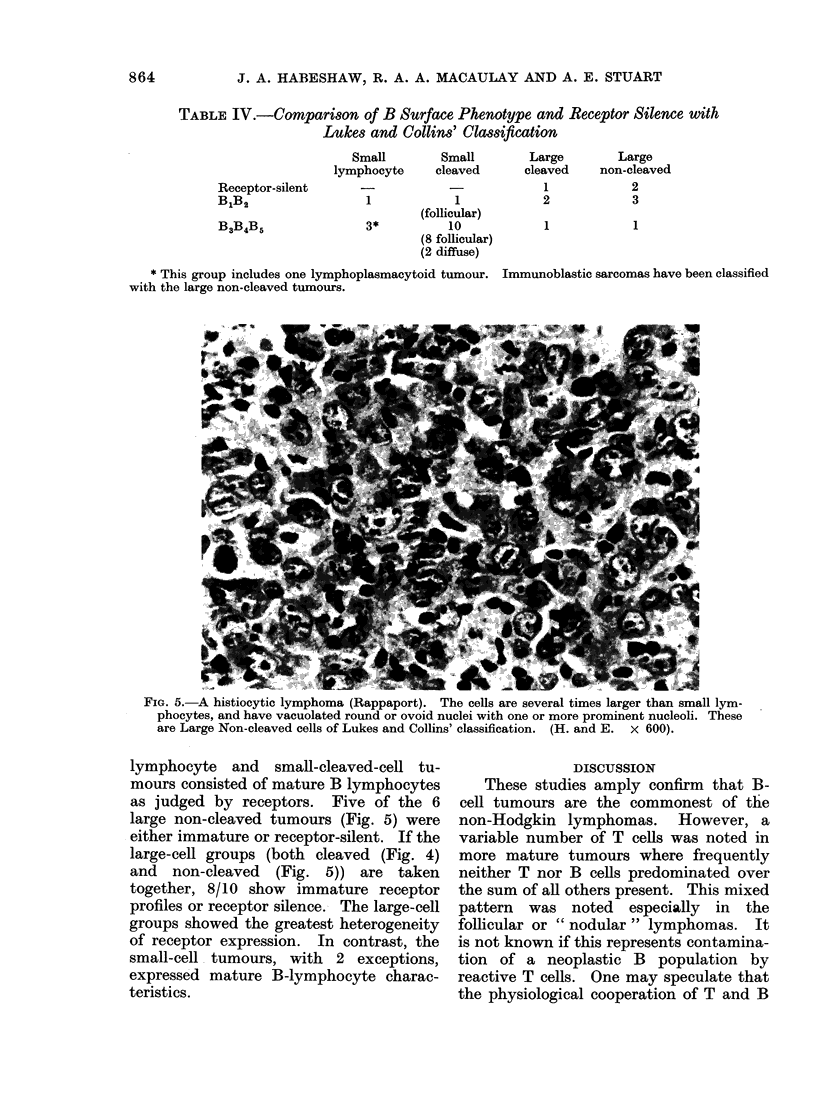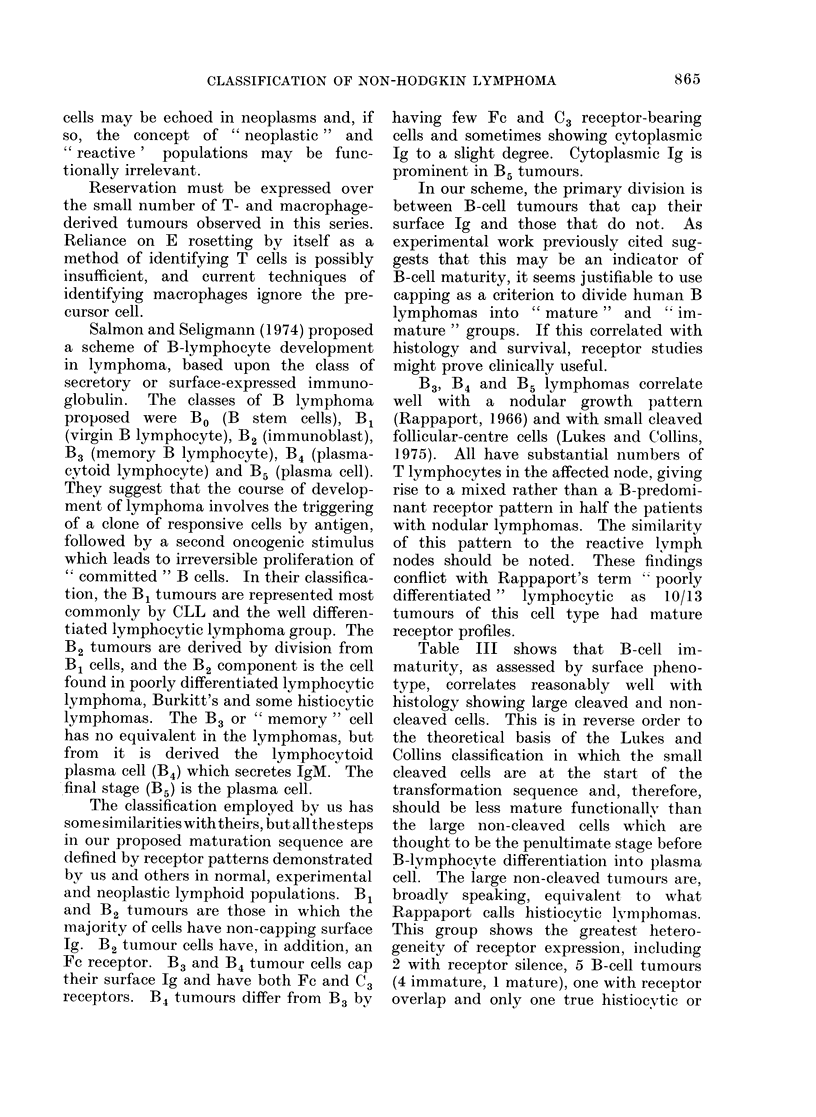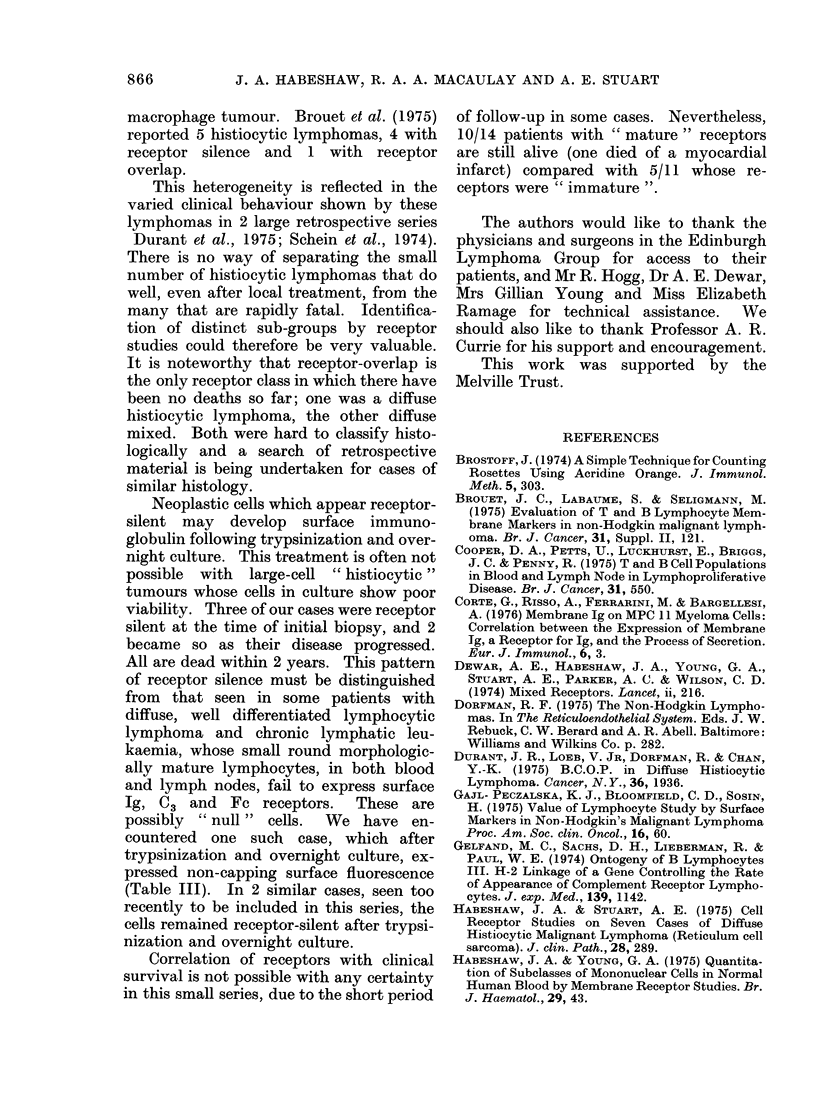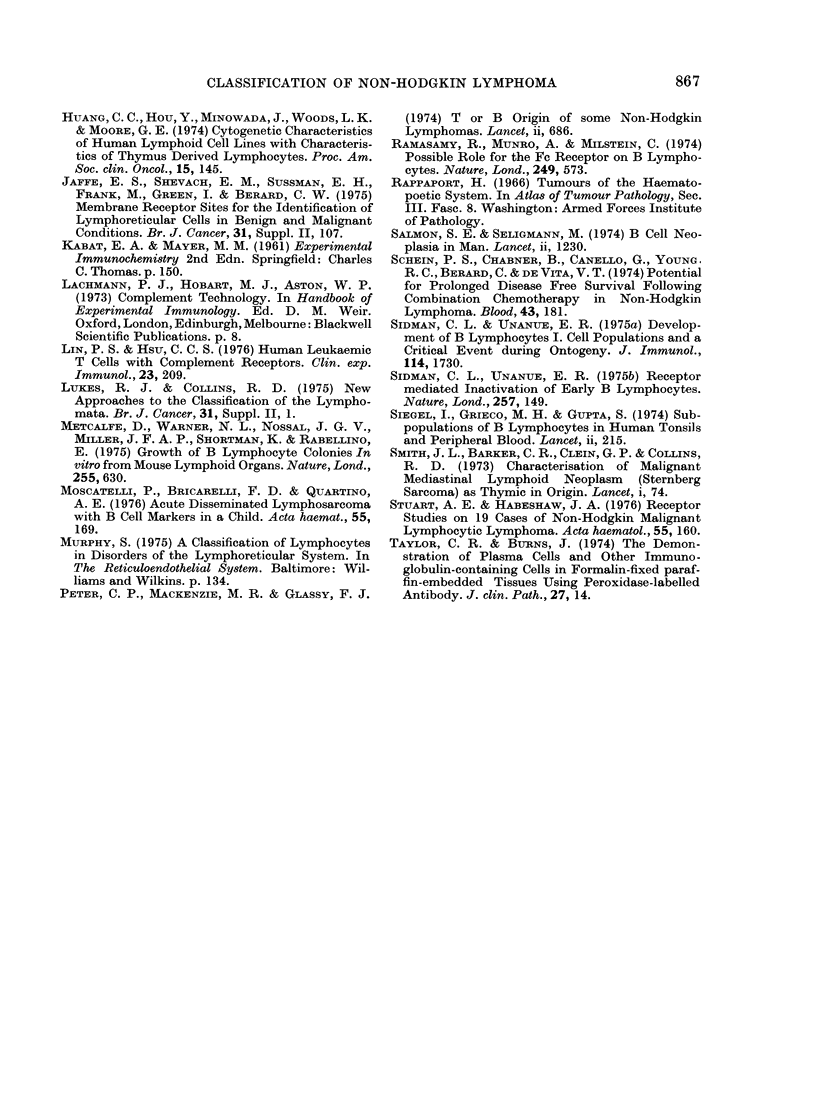# Correlation of surface receptors with histological appearance in 29 cases of non-Hodgkin lymphoma.

**DOI:** 10.1038/bjc.1977.129

**Published:** 1977-06

**Authors:** J. A. Habeshaw, R. A. Macaulay, A. E. Stuart

## Abstract

**Images:**


*Br. J. Cancer* (1977) **35**, 858

### CORRELATION OF SURFACE RECEPTORS WITH HISTOLOGICAL APPEARANCE IN 29 CASES OF NON-HODGKIN LYMPHOMA

J. A. HABESHAW, R. A. A. MACAULAY AND A. E. STUART

*From the Department of Pathology, University of Edinburgh Medical School, Teviot Place, Edinburgh EH8 9AG*

Received 2 August 1976 Accepted 28 January 1977

**Summary.—The receptor patterns of cell suspensions from 29 cases of non-Hodgkin lymphoma were correlated with the histology of the nodes from which the cells were taken. Twenty-two were judged to be predominantly or largely B-cell, and because of this preponderance these were divided by a method based on the distribution of surface immunoglobulin and the expression of Fc and $C_3$ receptors.**

**" Mature " B-cell and B-mixed tumours showing capping surface Ig with Fc and/or $C_3$ receptors correlated well with a nodular growth pattern, and consisted of what Rappaport (1966) calls "poorly differentiated" lymphocytes equivalent to the "small cleaved " cell as defined by Lukes and Collins (1975). Ten of the 14 patients in this receptor category are alive between 12 and 30 months after diagnosis.**

**Receptor-silent and " immature " B-cell tumours with non-capping surface Ig correlated predominantly with the Rappaport histiocytic lymphoma and Lukes and Collins' large cleaved and large non-cleaved lymphomas, though these histological categories also included a wide variety of other receptor types such as T-cell, Receptor-overlap and the single true Macrophage tumour. Five of the 11 patients with receptor-silent or immature B-cell tumours are alive between 7 and 15 months after the diagnosis.**

**Diffuse mixed and diffuse poorly differentiated lymphocytic lymphomas in Rappaport's classification correlated poorly with receptors, mature and immature B-cell tumours being equally represented.**

THE first purpose of this paper is to show how receptor studies on human lymphomas may be used to identify the cells present (Table I; Fig. 1). The second is to assess the maturity of the B cell by a number of cell surface markers, and the third is to relate the surface markers to the histology of the tumour. Previous attempts to identify the cells present in human lymphomas by surface marker techniques (Brouet, Labaume and Seligmann, 1975; Cooper *et al.*, 1975; Dorfman, 1975; Gajl-Peczalska, Bloomfield and Sosin, 1975; Habeshaw and Stuart, 1975; Jaffe *et al.*, 1975; Huang *et al.*, 1974; Peter, Mackenzie and Glassy, 1974; Stuart and Habeshaw, 1976; Smith *et al.*, 1973) have all shown that most non-Hodgkin lymphomas consist predominantly of B cells, with a minority of T cells. A few are derived from T cells, and those arising from macrophages are distinctly uncommon. Tumours without B, T or macrophage markers we call " receptor-silent ". Some authors have encountered tumours in which the sum of B and T cells appears to exceed the total cell count. This suggests simultaneous expression by cells of both B and T characteristics, named by us as " receptor overlap " (Lin and Hsu, 1976; Habeshaw and Stuart, 1975; Murphy, 1975).

The receptors expressed at different stages of B-cell differentiation in the mouse have been well documented (Gelfand *et al.*, 1974; Metcalf *et al.*, 1975;

Ramasamy, Munro and Milstein, 1974; Sidman and Unanue, 1975*a*, *b*). These showed failure of surface Ig to " cap " (aggregate at one pole of the cell) in the most immature stages. As maturation proceeds, the cells " cap " surface Ig and express first Fc and then both Fc and $C_3$ receptors. The plasma cells, or mature secretory B cells, have inconsistent surface markers, but contain intracytoplasmic Ig (Corte *et al.*, 1976). We and others have found identical variations in the pattern of surface Ig, Fc and $C_3$ receptors in human material such as normal peripheral blood (Habeshaw and Young, 1975), tonsil (Siegel, Grieco and Gupta, 1974), and in lymphomas (Brouet *et al.*, 1975; Dorfman, 1975; Jaffe *et al.*, 1975; Stuart and Habeshaw, 1976; Habeshaw and Stuart, 1975; Moscatelli, Bricarelli and Quartino, 1976). It therefore seems likely that the sequence of phenotype change seen in mature mouse B-lymphoid populations also occurs in man. If this were true, the expression of surface Ig, Fc and $C_3$ receptors may be a means of assessing cellular differentiation in human B-cell neoplasms. In this paper we propose a scheme of B-cell differentiation in human non-Hodgkin lymphoma based on the experimental and natural observations previously cited.

Tumours in which the B cells express an undifferentiated receptor profile should appear immature histologically and behave aggressively clinically. The opposite should apply to those tumours assessed as well differentiated by surface markers. In this paper we test this hypothesis in 25 tumours of B-cell or receptor-silent type.

### MATERIALS AND METHODS

The techniques employed are described in outline only, since details have been reported previously (Habeshaw and Young, 1975; Stuart and Habeshaw, 1976; Dewar *et al.*, 1975).

*Lymph-node cell suspensions.*—Cell suspensions in tissue culture medium were prepared from lymph-node biopsy specimens. Cells were not fractionated, and when viability was $< 60\%$, the suspension was discarded. The material is not selected in any other way than this, and represents a continuous series of cases.

*Antibodies: rabbit anti-sheep-red-cell and rabbit anti-ox-red-cell antibodies.*—IgM antibodies were prepared by the i.v. immunization of rabbits with ox or sheep erythrocyte stroma according to the method of Kabat and Mayer (1961). The IgM was obtained by Sephadex G 200 gel chromatography in 0·1M Tris/HCl or glycine buffer at pH 8·0–8·4. The first peak effluent contained most of the IgM and most antibody activity by tray titration.

Rabbit anti-ox-red-cell, anti-sheep-red-cell, and anti-human-red-cell IgG antibodies were made by conventional s.c. and i.p. immunization of rabbits, with the appropriate stroma. Purified IgG was obtained by salting out, dialysis against distilled water, and batch chromatography on DEAE cellulose.

Rabbit anti-human-$C_3$ and -$C_4$ antisera were used to demonstrate complement components on the surface of sensitized red cells.

*Formation of rosettes.*—In 23 cases, particles were prepared, and rosettes formed according to the standard methods described by Habeshaw and Young (1975). In 6 cases, Fc and $C_3$ receptors were prepared using rabbit anti-ox-RBC IgG (for Fc) and rabbit anti-ox-RBC IgM + human $R_3$ reagent ($C_3d$ receptor) (Lachman, Hobart and Aston, 1973). Controls showed that results obtained with sheep or ox red cells were comparable for Fc and $C_3d$ receptors. A heavily sensitized red cell is needed to detect *all* cells expressing an Fc receptor. Mixed C and Fc receptors were detected using human red cells sensitized with rabbit anti-human-RBC IgG as previously described (Dewar *et al.*, 1974). Rosette counting is facilitated by the addition of 0·1 ml of 0·01% solution of acridine orange to the suspended rosettes immediately before examination by fluorescence and phase-contrast microscopy (Brostoff, 1974).

*Immunofluorescent staining.*—Antibodies against human serum immunoglobulin were obtained commercially. These included polyvalent rabbit anti-human-immunoglobulin (Nordic), FITC-coupled goat anti-rabbit serum (Meloy, Nordic), and monospecific antisera against heavy (IgM, IgA, IgG, IgD) and light (Kappa, Lambda) chains of immunoglobulins (Nordic, Meloy, Dakopats). Capping is a phenomenon dependent upon the degree of cross-linking in the surface-Ig–anti-Ig complex, and both temperature and time of incubation. The conditions cited are those which specifically favour the formation of " caps " in normal B lymphoid tissue. Increasing the times of incubation beyond 30 min can lead to cap shedding or interiorization in normal lymphoid cells, and give rise to an erroneous measurement of B lymphoid cell numbers.

All antisera were centrifuged at 110,000 *g* for 1 h before use, to remove aggregates of Ig. They were then aliquoted in 0·1-ml quantities and stored at − 20°C for up to one month. It should be emphasized that removal of aggregated Ig from anti-human or anti-rabbit immunoglobulin antisera by high speed centrifugation is essential—otherwise B cells as well as all other cells expressing the Fc receptor will be stained, and any capping which occurs is then due to capping of Fc receptors rather than capping of surface Ig/anti Ig complex.

In 2 cases, the capacity of the tumour cells to synthesize surface Ig following trypsinization was assessed. Cells were treated with 0·25% trypsin (Armour) in PBS for 30 min at 37°C, to abolish surface Ig staining entirely. After washing, the cells were cultured in Medium 199 + 10% FCS for 24 h and reassessed for presence of surface Ig, and for cytoplasmic Ig.

*Receptor terminology.*—Using these methods, the tumours are assigned to the classes indicated in Tables I and II according to the receptor pattern that predominates over the sum of all the others.

The term " Mixed " implies that no single class exceeded the sum of all the others. In each of these cases, the word "mixed" is preceded by the largest single cell class.

*Receptor overlap.*—The sensitization procedure controls described by Dewar *et al.* (1974) ensure that single cells expressing both T and B characteristics are not overlooked.

*Receptor silence.*—Cells failing to express receptors by any of the methods outlined are clearly of unknown origin, and may be derived from a metastatic carcinoma or sarcoma in a lymph node. Where this has seemed likely on clinical and histological grounds, the case has been discarded, leaving, nonetheless, some cases of histologically undifferentiated neoplasms with an undoubted lymphomatous look, and clinical findings appropriate to that diagnosis.

*Histological and cytological evaluation.*—Tissue for histology was taken from the slice of lymph node biopsy next to that from which the cell suspensions were made. Fixation was in 10% formaldehyde in saline. Sections were cut at 2·5 μm and stained with H. and E., Gordon and Sweet's reticulin, Giemsa and methyl green–Pyronin stains. The tumours were classified using the Rappaport (1966) and Lukes and Collins (1975) classifications.

Dabs of freshly cut lymph-node surface and smears of the cell suspensions were stained with May–Grünwald–Giemsa. As well as giving increased cytological detail of the cells, these techniques helped us judge whether the cell population in the tissue sections was equivalent to that in the suspensions. Cytoplasmic immunoglobulin content of the tumours was assessed by the P.A.P. immunoglobulin technique of Taylor and Burns (1974).

### RESULTS

### *Receptor analysis*

The results of the receptor analysis in Table I show that 22/29 tumours were predominantly B-cell. Nine of these 22 were classed as " B mixed " since they contained an appreciable number of T cells. Two showed receptor overlap, one was a macrophage tumour and the last a T-cell tumour.

Table II shows how the B lymphomas can be further subdivided according to their expression of surface Ig, Fc and $C_3$ receptors and cytoplasmic Ig. Although the origin of receptor-silent cells is unknown, they are included, for convenience, with the B cells. The horizontal line indicates an important division between tumours whose cells cap their surface Ig ($B_3B_4B_5$), called mature, and those that do not ($B_1B_2$), regarded as immature. Mature cells express receptors for Fc and $C_3$, whilst immature cells may have no surface receptors or an Fc receptor only, but never both Fc and $C_3$. Cases expressing only $C_3$ receptors on non-capping B cells were not seen in this series, but may well occur. Capping of

TABLE I.—*Scheme of Receptor Expression in Non-Hodgkin Lymphoma*

| Category | Characteristics: E Rosette | $C_3$ | Fc | Phagocytosis of Neutral Red | Surface Ig | Number of cases in present series |
|---|---|---|---|---|---|---|
| B Lymphoma | — | +/- | +/- | — | ++ | 22 |
| T Lymphoma | ++ | — | — | — | — | 1 |
| Macrophage | — | + | ++ | ++ | — | 1 |
| Receptor-silent | — | — | — | — | — | 3 |
| Receptor overlap | ++ | ++/- | + | — | ++/- | 2 |

\+ Shown by a substantial number of lymph-node cells.
\++ Shown by the majority of lymph-node cells.
— Not shown by lymph-node cells.

TABLE II.—*Sub-division of B-cell Lymphomas on the Basis of Surface Phenotype and Functional Attributes of the Neoplastic Cells*

| Category | Characteristics: Surface Ig | Fc | $C_3$ | Cytoplasmic Ig | Number of cases |
|---|---|---|---|---|---|
| Receptor-silent | — | — | — | — | 3 |
| $B_1$ (Immature) | Non-capping | — | — | — | 5 |
| $B_2$ (Immature) | Non-capping | + | — | — | 3 |
| $B_3$ (Mature) | Capping | ++ | ++ | — | 9 |
| $B_4$ (Mature) | Capping | + | + | ± | 4 |
| $B_5$ (Mature) | — | — | ± | +++ | 1 |
| | | | | | 25 |

— Not shown by B cells.
\+ Shown by a substantial number of B cells.
\++ Shown by the majority of B cells.
\+++ Shown by all B cells.

FIG. 1.—Capping and non-capping surface-Ig-bearing cells in 28 control lymph nodes (▲) and 22 B-cell or mixed lymphomas (○).

surface immunoglobulin (Fig. 1) occurs more commonly in reactive than in neoplastic nodes. Nevertheless, a higher proportion of non-capping cells was found in 2 highly reactive nodes with germinal centres giving a $B_2$ receptor profile. Eighty per cent of the control lymph nodes gave a mixed receptor pattern.

*Comparison of receptor patterns with Rappaport's histological classification* (1966)

The correlation of histology with the B surface phenotype is shown in Table III. Three of the 4 well differentiated lymphocytic lymphomas (Fig. 2) had cells that were mature both morphologically and by their expressed receptors. Two of these are alive and in remission, the third, also in remission, died of a myocardial infarct. The fourth patient has clinical features typical of chronic lymphocytic leukaemia but, unlike the other three, his lymphocytes, in both blood and lymph node biopsy, expressed an immature receptor pattern.

FIG. 2.—A well differentiated lymphocytic lymphoma (Rappaport). This is equivalent to Lukes and Collins' small lymphocytic lymphoma. (H. and E. × 600).

FIG. 3.—Cells from a nodule of poorly differentiated lymphocytic lymphoma (Rappaport). Nuclei are slightly larger than those of well differentiated lymphocytes and have irregular outlines, often with conspicuous clefts. These would be classified as Small Cleaved Follicular-centre cells by Lukes and Collins. (H. and E. × 600).

FIG. 4.—The cells of this poorly differentiated lymphocytic lymphoma are larger than those in Figure 5, but maintain their irregular and cleaved nuclear outline. Nucleoli indistinct. In Lukes and Collins' classification these would be termed Large Cleaved Follicular-centre cells. (H. and E. × 600).

TABLE III.—*Comparison of B Surface Phenotype and Receptor Silence with Rappaport's Classification*

| | DWL | NPL | DM DPL | DH |
|---|---|---|---|---|
| Receptor-silent | — | — | 1 | 2 |
| $B_1B_2$ | 1 | 1 | 2 | 4 |
| $B_3B_4B_5$ | 3 | 8 | 2 | 1 |

D = Diffuse. W = Well differentiated.
N = Nodular. P = Poorly differentiated.
L = Lymphocytic. M = Mixed.
H = Histiocytic or undifferentiated.

In the nodular lymphomas, all were poorly differentiated lymphocytic histologically (Figs. 3 and 4), all but one showed a mature receptor pattern and all but one are alive and in remission. There was a sequential change in the receptors of the patient who died. At biopsy, the lymph node cells were $B_3$ (with a high proportion T cells), but the blood B lymphocytes were $B_2$, with a receptor-silent population of 10%. In spite of histology that frequently indicates a good prognosis, the patient had rapidly progressive disease and 10 days later the receptor-silent population in the blood had risen to 40%. The patient died soon after, and autopsy showed a diffuse poorly differentiated lymphocytic lymphoma. We have since encountered a case of follicular (nodular) lymphoma where the follicle-centre cells were unusually large and there were numerous mitotic figures. The receptor profile was $B_2$.

In the diffuse poorly differentiated and diffuse mixed group there were tumours with both mature and immature B receptor patterns and poor correlation with histology. The Rappaport " Histiocytic " group included 5 B-cell tumours and 2 with receptor silence. Four of the B-cell tumours had an immature receptor pattern. One patient with a $B_1$ tumour became leukaemic terminally, with the majority of his peripheral blood cells receptor-silent.

*Comparison of receptor patterns with Lukes and Collins' classification* (1975)

Table IV shows that 13/15 small-

TABLE IV.—*Comparison of B Surface Phenotype and Receptor Silence with Lukes and Collins' Classification*

| | Small lymphocyte | Small cleaved | Large cleaved | Large non-cleaved |
|---|---|---|---|---|
| Receptor-silent | — | — | 1 | 2 |
| $B_1B_2$ | 1 | 1 (follicular) | 2 | 3 |
| $B_3B_4B_5$ | 3* | 10 (8 follicular) (2 diffuse) | 1 | 1 |

* This group includes one lymphoplasmacytoid tumour. Immunoblastic sarcomas have been classified with the large non-cleaved tumours.

FIG. 5.—A histiocytic lymphoma (Rappaport). The cells are several times larger than small lymphocytes, and have vacuolated round or ovoid nuclei with one or more prominent nucleoli. These are Large Non-cleaved cells of Lukes and Collins' classification. (H. and E. × 600).

lymphocyte and small-cleaved-cell tumours consisted of mature B lymphocytes as judged by receptors. Five of the 6 large non-cleaved tumours (Fig. 5) were either immature or receptor-silent. If the large-cell groups (both cleaved (Fig. 4) and non-cleaved (Fig. 5)) are taken together, 8/10 show immature receptor profiles or receptor silence. The large-cell groups showed the greatest heterogeneity of receptor expression. In contrast, the small-cell tumours, with 2 exceptions, expressed mature B-lymphocyte characteristics.

### DISCUSSION

These studies amply confirm that B-cell tumours are the commonest of the non-Hodgkin lymphomas. However, a variable number of T cells was noted in more mature tumours where frequently neither T nor B cells predominated over the sum of all others present. This mixed pattern was noted especially in the follicular or " nodular " lymphomas. It is not known if this represents contamination of a neoplastic B population by reactive T cells. One may speculate that the physiological cooperation of T and B

cells may be echoed in neoplasms and, if so, the concept of " neoplastic " and " reactive ' populations may be functionally irrelevant.

Reservation must be expressed over the small number of T- and macrophage-derived tumours observed in this series. Reliance on E rosetting by itself as a method of identifying T cells is possibly insufficient, and current techniques of identifying macrophages ignore the precursor cell.

Salmon and Seligmann (1974) proposed a scheme of B-lymphocyte development in lymphoma, based upon the class of secretory or surface-expressed immunoglobulin. The classes of B lymphoma proposed were $B_0$ (B stem cells), $B_1$ (virgin B lymphocyte), $B_2$ (immunoblast), $B_3$ (memory B lymphocyte), $B_4$ (plasmacytoid lymphocyte) and $B_5$ (plasma cell). They suggest that the course of development of lymphoma involves the triggering of a clone of responsive cells by antigen, followed by a second oncogenic stimulus which leads to irreversible proliferation of " committed " B cells. In their classification, the $B_1$ tumours are represented most commonly by CLL and the well differentiated lymphocytic lymphoma group. The $B_2$ tumours are derived by division from $B_1$ cells, and the $B_2$ component is the cell found in poorly differentiated lymphocytic lymphoma, Burkitt's and some histiocytic lymphomas. The $B_3$ or " memory " cell has no equivalent in the lymphomas, but from it is derived the lymphocytoid plasma cell ($B_4$) which secretes IgM. The final stage ($B_5$) is the plasma cell.

The classification employed by us has some similarities with theirs, but all the steps in our proposed maturation sequence are defined by receptor patterns demonstrated by us and others in normal, experimental and neoplastic lymphoid populations. $B_1$ and $B_2$ tumours are those in which the majority of cells have non-capping surface Ig. $B_2$ tumour cells have, in addition, an Fc receptor. $B_3$ and $B_4$ tumour cells cap their surface Ig and have both Fc and $C_3$ receptors. $B_4$ tumours differ from $B_3$ by having few Fc and $C_3$ receptor-bearing cells and sometimes showing cytoplasmic Ig to a slight degree. Cytoplasmic Ig is prominent in $B_5$ tumours.

In our scheme, the primary division is between B-cell tumours that cap their surface Ig and those that do not. As experimental work previously cited suggests that this may be an indicator of B-cell maturity, it seems justifiable to use capping as a criterion to divide human B lymphomas into " mature " and " immature " groups. If this correlated with histology and survival, receptor studies might prove clinically useful.

$B_3$, $B_4$ and $B_5$ lymphomas correlate well with a nodular growth pattern (Rappaport, 1966) and with small cleaved follicular-centre cells (Lukes and Collins, 1975). All have substantial numbers of T lymphocytes in the affected node, giving rise to a mixed rather than a B-predominant receptor pattern in half the patients with nodular lymphomas. The similarity of this pattern to the reactive lymph nodes should be noted. These findings conflict with Rappaport's term " poorly differentiated " lymphocytic as 10/13 tumours of this cell type had mature receptor profiles.

Table III shows that B-cell immaturity, as assessed by surface phenotype, correlates reasonably well with histology showing large cleaved and non-cleaved cells. This is in reverse order to the theoretical basis of the Lukes and Collins classification in which the small cleaved cells are at the start of the transformation sequence and, therefore, should be less mature functionally than the large non-cleaved cells which are thought to be the penultimate stage before B-lymphocyte differentiation into plasma cell. The large non-cleaved tumours are, broadly speaking, equivalent to what Rappaport calls histiocytic lymphomas. This group shows the greatest heterogeneity of receptor expression, including 2 with receptor silence, 5 B-cell tumours (4 immature, 1 mature), one with receptor overlap and only one true histiocytic or macrophage tumour. Brouet *et al.* (1975) reported 5 histiocytic lymphomas, 4 with receptor silence and 1 with receptor overlap.

This heterogeneity is reflected in the varied clinical behaviour shown by these lymphomas in 2 large retrospective series Durant *et al.*, 1975; Schein *et al.*, 1974). There is no way of separating the small number of histiocytic lymphomas that do well, even after local treatment, from the many that are rapidly fatal. Identification of distinct sub-groups by receptor studies could therefore be very valuable. It is noteworthy that receptor-overlap is the only receptor class in which there have been no deaths so far; one was a diffuse histiocytic lymphoma, the other diffuse mixed. Both were hard to classify histologically and a search of retrospective material is being undertaken for cases of similar histology.

Neoplastic cells which appear receptor-silent may develop surface immunoglobulin following trypsinization and overnight culture. This treatment is often not possible with large-cell " histiocytic " tumours whose cells in culture show poor viability. Three of our cases were receptor silent at the time of initial biopsy, and 2 became so as their disease progressed. All are dead within 2 years. This pattern of receptor silence must be distinguished from that seen in some patients with diffuse, well differentiated lymphocytic lymphoma and chronic lymphatic leukaemia, whose small round morphologically mature lymphocytes, in both blood and lymph nodes, fail to express surface Ig, $C_3$ and Fc receptors. These are possibly " null " cells. We have encountered one such case, which after trypsinization and overnight culture, expressed non-capping surface fluorescence (Table III). In 2 similar cases, seen too recently to be included in this series, the cells remained receptor-silent after trypsinization and overnight culture.

Correlation of receptors with clinical survival is not possible with any certainty in this small series, due to the short period of follow-up in some cases. Nevertheless, 10/14 patients with " mature " receptors are still alive (one died of a myocardial infarct) compared with 5/11 whose receptors were " immature ".

The authors would like to thank the physicians and surgeons in the Edinburgh Lymphoma Group for access to their patients, and Mr R. Hogg, Dr A. E. Dewar, Mrs Gillian Young and Miss Elizabeth Ramage for technical assistance. We should also like to thank Professor A. R. Currie for his support and encouragement.

This work was supported by the Melville Trust.

### REFERENCES

BROSTOFF, J. (1974) A Simple Technique for Counting Rosettes Using Acridine Orange. *J. Immunol. Meth.* **5**, 303.

BROUET, J. C., LABAUME, S. & SELIGMANN, M. (1975) Evaluation of T and B Lymphocyte Membrane Markers in non-Hodgkin malignant lymphoma. *Br. J. Cancer*, **31**, Suppl. II, 121.

COOPER, D. A., PETTS, U., LUCKHURST, E., BRIGGS, J. C. & PENNY, R. (1975) T and B Cell Populations in Blood and Lymph Node in Lymphoproliferative Disease. *Br. J. Cancer*, **31**, 550.

CORTE, G., RISSO, A., FERRARINI, M. & BARGELLESI, A. (1976) Membrane Ig on MPC 11 Myeloma Cells: Correlation between the Expression of Membrane Ig, a Receptor for Ig, and the Process of Secretion. *Eur. J. Immunol.*, **6**, 3.

DEWAR, A. E., HABESHAW, J. A., YOUNG, G. A., STUART, A. E., PARKER, A. C. & WILSON, C. D. (1974) Mixed Receptors. *Lancet*, ii, 216.

DORFMAN, R. F. (1975) The Non-Hodgkin Lymphomas. In *The Reticuloendothelial System*. Eds. J. W. Rebuck, C. W. Berard and A. R. Abell. Baltimore: Williams and Wilkins Co. p. 282.

DURANT, J. R., LOEB, V. JR, DORFMAN, R. & CHAN, Y.-K. (1975) B.C.O.P. in Diffuse Histiocytic Lymphoma. *Cancer, N.Y.*, **36**, 1936.

GAJL- PECZALSKA, K. J., BLOOMFIELD, C. D., SOSIN, H. (1975) Value of Lymphocyte Study by Surface Markers in Non-Hodgkin's Malignant Lymphoma *Proc. Am. Soc. clin. Oncol.*, **16**, 60.

GELFAND, M. C., SACHS, D. H., LIEBERMAN, R. & PAUL, W. E. (1974) Ontogeny of B Lymphocytes III. H-2 Linkage of a Gene Controlling the Rate of Appearance of Complement Receptor Lymphocytes. *J. exp. Med.*, **139**, 1142.

HABESHAW, J. A. & STUART, A. E. (1975) Cell Receptor Studies on Seven Cases of Diffuse Histiocytic Malignant Lymphoma (Reticulum cell sarcoma). *J. clin. Path.*, **28**, 289.

HABESHAW, J. A. & YOUNG, G. A. (1975) Quantitation of Subclasses of Mononuclear Cells in Normal Human Blood by Membrane Receptor Studies. *Br. J. Haematol.*, **29**, 43.

HUANG, C. C., HOU, Y., MINOWADA, J., WOODS, L. K. & MOORE, G. E. (1974) Cytogenetic Characteristics of Human Lymphoid Cell Lines with Characteristics of Thymus Derived Lymphocytes. *Proc. Am. Soc. clin. Oncol.*, **15**, 145.

JAFFE, E. S., SHEVACH, E. M., SUSSMAN, E. H., FRANK, M., GREEN, I. & BERARD, C. W. (1975) Membrane Receptor Sites for the Identification of Lymphoreticular Cells in Benign and Malignant Conditions. *Br. J. Cancer*, **31**, Suppl. II, 107.

KABAT, E. A. & MAYER, M. M. (1961) *Experimental Immunochemistry* 2nd Edn. Springfield: Charles C. Thomas. p. 150.

LACHMANN, P. J., HOBART, M. J., ASTON, W. P. (1973) Complement Technology. In *Handbook of Experimental Immunology*. Ed. D. M. Weir. Oxford, London, Edinburgh, Melbourne: Blackwell Scientific Publications. p. 8.

LIN, P. S. & HSU, C. C. S. (1976) Human Leukaemic T Cells with Complement Receptors. *Clin. exp. Immunol.*, **23**, 209.

LUKES, R. J. & COLLINS, R. D. (1975) New Approaches to the Classification of the Lymphomata. *Br. J. Cancer*, **31**, Suppl. II, 1.

METCALFE, D., WARNER, N. L., NOSSAL, J. G. V., MILLER, J. F. A. P., SHORTMAN, K. & RABELLINO, E. (1975) Growth of B Lymphocyte Colonies *In vitro* from Mouse Lymphoid Organs. *Nature, Lond.*, **255**, 630.

MOSCATELLI, P., BRICARELLI, F. D. & QUARTINO, A. E. (1976) Acute Disseminated Lymphosarcoma with B Cell Markers in a Child. *Acta haemat.*, **55**, 169.

MURPHY, S. (1975) A Classification of Lymphocytes in Disorders of the Lymphoreticular System. In *The Reticuloendothelial System*. Baltimore: Williams and Wilkins. p. 134.

PETER, C. P., MACKENZIE, M. R. & GLASSY, F. J. (1974) T or B Origin of some Non-Hodgkin Lymphomas. *Lancet*, ii, 686.

RAMASAMY, R., MUNRO, A. & MILSTEIN, C. (1974) Possible Role for the Fc Receptor on B Lymphocytes. *Nature, Lond.*, **249**, 573.

RAPPAPORT, H. (1966) Tumours of the Haematopoetic System. In *Atlas of Tumour Pathology*, Sec. III. Fasc. 8. Washington: Armed Forces Institute of Pathology.

SALMON, S. E. & SELIGMANN, M. (1974) B Cell Neoplasia in Man. *Lancet*, ii, 1230.

SCHEIN, P. S., CHABNER, B., CANELLO, G., YOUNG, R. C., BERARD, C. & DE VITA, V. T. (1974) Potential for Prolonged Disease Free Survival Following Combination Chemotherapy in Non-Hodgkin Lymphoma. *Blood*, **43**, 181.

SIDMAN, C. L. & UNANUE, E. R. (1975*a*) Development of B Lymphocytes I. Cell Populations and a Critical Event during Ontogeny. *J. Immunol.*, **114**, 1730.

SIDMAN, C. L., UNANUE, E. R. (1975*b*) Receptor mediated Inactivation of Early B Lymphocytes. *Nature, Lond.*, **257**, 149.

SIEGEL, I., GRIECO, M. H. & GUPTA, S. (1974) Subpopulations of B Lymphocytes in Human Tonsils and Peripheral Blood. *Lancet*, ii, 215.

SMITH, J. L., BARKER, C. R., CLEIN, G. P. & COLLINS, R. D. (1973) Characterisation of Malignant Mediastinal Lymphoid Neoplasm (Sternberg Sarcoma) as Thymic in Origin. *Lancet*, i, 74.

STUART, A. E. & HABESHAW, J. A. (1976) Receptor Studies on 19 Cases of Non-Hodgkin Malignant Lymphocytic Lymphoma. *Acta haematol.*, **55**, 160.

TAYLOR, C. R. & BURNS, J. (1974) The Demonstration of Plasma Cells and Other Immunoglobulin-containing Cells in Formalin-fixed paraffin-embedded Tissues Using Peroxidase-labelled Antibody. *J. clin. Path.*, **27**, 14.